# Airborne Pesticides—Deep Diving into Sampling and Analysis

**DOI:** 10.3390/toxics11110883

**Published:** 2023-10-27

**Authors:** Patchimaporn Udomkun, Thirasant Boonupara, Sulak Sumitsawan, Eakalak Khan, Siwatt Pongpichan, Puangrat Kajitvichyanukul

**Affiliations:** 1 Department of Environmental Engineering, Faculty of Engineering, Chiang Mai University, Chiang Mai 50200, Thailand; udomkun.patchimaporn@gmail.com (P.U.); jarmore001@gmail.com (T.B.); or sulak.sumit@gmail.com (S.S.); 2 Office of Research Administration, Chiang Mai University, Chiang Mai 50200, Thailand; 3 Civil and Environmental Engineering and Construction Department, University of Nevada, Las Vegas, NV 89154-4015, USA; eakalak.khan@unlv.edu; 4 NIDA Center for Research and Development of Disaster Prevention and Management, Graduate School of Social Development and Management Strategy, National Institute of Development Administration (NIDA), Bangkok 10240, Thailand

**Keywords:** pesticide analysis, atmospheric contaminants, airborne sampling, extraction approaches, analytical modalities, computational modeling

## Abstract

The escalating utilization of pesticides has led to pronounced environmental contamination, posing a significant threat to agroecosystems. The extensive and persistent global application of these chemicals has been linked to a spectrum of acute and chronic human health concerns. This review paper focuses on the concentrations of airborne pesticides in both indoor and outdoor environments. The collection of diverse pesticide compounds from the atmosphere is examined, with a particular emphasis on active and passive air sampling techniques. Furthermore, a critical evaluation is conducted on the methodologies employed for the extraction and subsequent quantification of airborne pesticides. This analysis takes into consideration the complexities involved in ensuring accurate measurements, highlighting the advancements and limitations of current practices. By synthesizing these aspects, this review aims to foster a more comprehensive and informed comprehension of the intricate dynamics related to the presence and measurement of airborne pesticides. This, in turn, is poised to significantly contribute to the refinement of environmental monitoring strategies and the augmentation of precise risk assessments.

## 1. Introduction

Acute or chronic exposure to airborne pesticides has the potential to negatively affect human health. Some pesticides are known to be toxic, and long-term exposure to low levels of them has been associated with adverse health effects, including respiratory issues, neurodevelopmental disorders, hormonal disruption, and certain types of cancers [1,2,3,4]. Furthermore, the environmental impact of airborne pesticides extends to non-target organisms, including wildlife, beneficial insects, and aquatic ecosystems [5,6].

Accurately measuring pesticides in the air is of paramount importance. However, routine monitoring and reporting of these pesticides in the air are lacking. Furthermore, analyzing these pesticides in both indoor and outdoor air presents complexities that require specific procedures. Various techniques and methods are used for this purpose. For example, active sampling methods involve actively pulling air through samplers using pumps or fans [7], while passive sampling methods rely on the diffusion of pesticides into a sorbent material [8].

After sample collection, extraction techniques are employed to isolate the pesticide compounds from the collected air samples. Common extraction methods include solid-phase extraction (SPE), liquid-liquid extraction (LLE), and solid-phase microextraction (SPME). With the increasing focus on miniaturization and the adoption of green chemistry principles, environmentally friendly sample preparation methods have gained popularity for pesticide extraction. One such method is pressurized liquid extraction (PLE), which offers high efficiency and low consumption of organic solvents for extracting pesticides from particulate matter (PM) [9]. Microwave-assisted extraction (MAE) is another technique that has been utilized for pesticide extraction, offering similar advantages [10]. In contrast, miniaturized systems based on ultrasonic-assisted extraction (UAE) have emerged as a simple and cost-effective alternative for extracting pollutants from PM [11]. Moreover, different solvents and sorbents can be utilized depending on the properties of the target pesticides. Analytical methods such as gas chromatography (GC), liquid chromatography (LC), or their combination with mass spectrometry (MS) are commonly employed for the analysis and quantification of airborne pesticides [12]. In addition to the traditional methods, alternative techniques like ion mobility spectrometry (IMS) and attenuated total reflectance–Fourier-transform infrared spectroscopy (ATR-FTIR) have been applied to measure airborne pesticides [13,14]. These methods allow for the identification and quantification of specific pesticide compounds present in the samples. Additionally, high-resolution mass spectrometry (HRMS) techniques offer improved sensitivity and selectivity in pesticide analysis [15].

This study carried out a comprehensive literature review in adherence to the Preferred Reporting Items for Systematic Reviews and Meta-Analyses (PRISMA) guidelines [16]. A systematic search was conducted utilizing a range of search terms within the ScienceDirect and PubMed databases. Inclusion criteria were limited to articles published in the English language, with a primary emphasis on recent research ranging from 2005 to 2023. The review focuses on two key aspects. Firstly, levels of pesticide concentrations in indoor and outdoor air: the review examines the presence and concentrations of pesticides in both indoor and outdoor air, shedding light on the extent of pesticide pollution in different environments. Secondly, analytical methodologies for pesticide analysis: various sampling, extraction, and analysis techniques employed in pesticide analysis are explored. The review critically discusses these methodologies in detail, including recommendations for suitable commercial materials such as adsorbents and filters. The objective of this review is to advance comprehension regarding the detection and analysis of airborne pesticides, contributing to more robust monitoring approaches and improved risk assessments.

## 2. Pesticide Concentrations in Air

### 2.1. Indoor Air

The potential adverse health effects stemming from pesticide exposure have garnered substantial public attention. While numerous studies have extensively examined and reported on their presence in water and soil, less focus has been directed towards their occurrence in the air. This is chiefly due to the fact that pesticides generally exist in the air as trace-level pollutants, ranging from picograms per cubic meter (pg m^−3^) to nanograms per cubic meter (ng m^−3^). Pesticides can manifest in the air in diverse states, encompassing solids, gases, and liquids [17]. Agricultural spraying activities, for instance, contribute to around 30–50% dispersion of most pesticides into the air [18], primarily facilitated through drift (wind-carried) and evaporation. Subsequently, pesticides undergo volatilization from soil and plants, degradation, and photolysis, eventually integrating into the atmospheric environment. Airborne pesticides present a dual concern, as they not only pose immediate health risks through inhalation but can also settle on surfaces, leading to prolonged indoor contamination. Some studies have consistently highlighted the prevalence of organochlorine and organophosphate pesticides as the prominent groups detected in indoor air (Table 1). Within the organochlorine group, compounds like dichlorodiphenyldichloroethylene (4,4′-DDE), dichlorodiphenyltrichloroethane (4,4′-DDT), hexachlorobenzene (HCB), hexachlorocyclohexane (HCH), and endosulfan have frequently emerged as detectable entities. Their concentrations ranged from 1.2 to 190 ng m^−3^, not detected to 5.0 ng m^−3^, 0.2 to 1.8 ng m^−3^, 0.1 to 7.8 ng m^−3^, and 0.3 to 0.8 ng m^−3^, respectively. Meanwhile, in the realm of organophosphates, chlorpyriphos, diazinon, and malathion have garnered significant attention, often being prominently identified. Their concentrations exhibited a range from 0.4 to 83.4 ng m^−3^ for chlorpyriphos, 1.0 to 427.5 ng m^−3^ for diazinon, and 0.2 to 16.1 ng m^−3^ for malathion.

Studies have examined the temporal analysis of pesticide residues to pinpoint factors influencing the transportation and redistribution of these compounds within indoor environments. For example, Obendorf et al. [40] discovered that the occurrence and quantity of pesticide residues, particularly chlorpyrifos, were elevated during the summer months—a correlation attributed to agricultural and horticultural practices. Conversely, greater quantities of insecticides like mecoprop, resmethrin, and tetramethrin were detected on flat surfaces during the winter, suggesting household application and potential redistribution within the indoor environment. In addition, a study conducted by Berger-Preiss and Elflein [41] observed the presence of pesticides (such as pyrethroids, pyrethrum, and the synergist piperonyl butoxide) in an experimental house over a two-year period. The concentrations of these pesticides were the highest at the top of the room and gradually decreased towards the middle and lower areas [42]. While some pesticides may degrade quickly through photodecomposition, others like DDT, chlordane, heptachlor, methoxychlor, dieldrin, and pentachlorophenol tend to persist indoors [43]. Lee et al. [35] also investigated the extent of pesticide residue contamination indoors. Notably, they observed that dichlorvos was present at higher levels, while chlorpyrifos and cypermethrin were detected at comparatively lower concentrations. Furthermore, they documented a decline in the concentrations of pesticide residues within indoor air over time. Specifically, the concentration of dichlorvos decreased from 0.458 to 0.073 mg/m³ over a 4-week period. Similarly, the levels of other substances declined from below 0.050 mg/m³ to non-detectable levels within just 2 weeks. 

Household pesticides are also found in dust and PM. The arena of dust and suspended particles becomes another tableau for household pesticides, as Rudel et al. [43] revealed the prevalence of permethrins and the synergist piperonyl butoxide, woven into concentrations ranging from 1.7 to 17 µg g^−1^. Wang et al. [44] gathered dust samples from floors within residential homes and office spaces. Among the array of pesticides, it was observed that hexachlorobenzene’s presence in indoor settings could be attributed to its transfer from outdoor sources [45]. On the other hand, chlordanes were linked to historical household usage [46], while pyrethroids showed an association with ongoing household applications [47]. The complex interplay between airborne pesticides and their residues highlights the intricate pathways through which these chemicals infiltrate indoor environments. These insights play a pivotal role in achieving a comprehensive grasp of exposure dynamics, thereby facilitating the formulation of focused strategies for effective monitoring, management, and the protection of human health.

### 2.2. Outdoor Air

Various pesticide groups, including organochlorine insecticides, and organophosphate insecticides, herbicides, and fungicides, have been detected in outdoor air samples from several countries such as France, Spain, China, Pakistan, Malaysia, South Africa, and the United States. Table 1 provides an overview of the concentration ranges of pesticides found in outdoor air. Mirroring the trend observed with indoor pesticides, the organochlorine insecticides that have consistently surfaced as the most frequently detected compounds over the past 15 years include 4,4′-DDE, 4,4′-DDD, and endosulfan. Their concentrations ranged from 0.002 to 25.6 ng m^−3^, 0.011 to 154 ng m^−3^, and 0.0001 to 81.3 ng m^−3^, respectively. Much like indoor environments, outdoor air contains traces of organophosphate insecticides such as chlorpyrifos, malathion, and diazinon, although typically at lower concentrations. However, their concentrations are commonly higher in indoor air compared to outdoor air due to their prevalent use for indoor pest control. Factors such as their proximity to indoor application sources, limited dispersion indoors, volatility, persistence, surface deposition, and potential re-suspension contribute to their higher concentrations. Outdoor air, in contrast, experiences lower exposure to these compounds due to differences in usage patterns and dispersion dynamics. 

It has been found that various herbicides and fungicides are notably present in fine airborne PM_2.5_, as highlighted in studies by Coscollà et al. [31,48], as well as by Sarigiannis et al. [36]. This finding is of particular significance as it offers insights into the capacity of these compounds to become airborne and potentially be transported across greater distances, facilitated by their association with finer PM. Consequently, in order to enhance a comprehension of the prevalence and behaviors of these pesticides, ongoing initiatives for their monitoring have been set in motion [31]. These monitoring efforts encompass a wide range of meticulously designed techniques and methodologies aimed at collecting, identifying, and quantifying the presence of these substances in the atmosphere. A detailed exploration of these techniques is presented in the upcoming section.

## 3. Advancements in Pesticide Sampling Techniques: Current Technology and Limitations

Under the scenario that only 10% of the pesticides applied to crops reaches the target organism, this means that 90% of them is deposited in non-target compartments [49]. Subsequently, these pesticides can contaminate the soil, air, surface water and groundwater, and sediments, as well as agricultural products via adsorption, leaching, volatilization, spray drift, and runoff processes [50]. In addition, different types of pesticides lead to different impacts on both environmental components and human health.

### 3.1. Active Sampling Applications and Limitations

**Figure 1:**
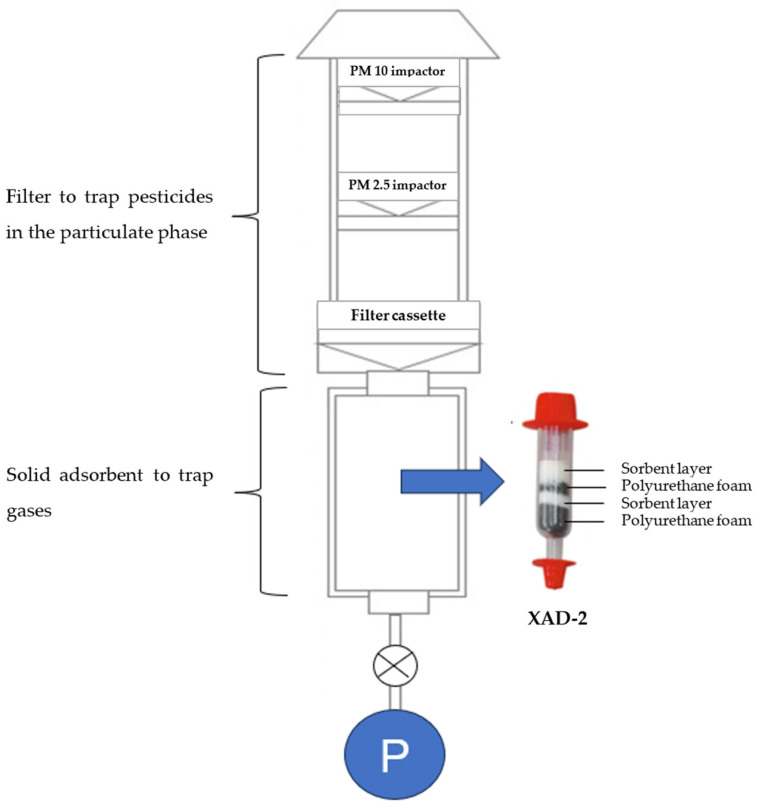
Diagram illustrating the active air sampling technique.

Active air sampling (AAS) is a technique that involves using a pump to collect gases, vapors, and particulates in a tube equipped with a sorbent bed or a size-selective sampler with a filter [51,52] (Figure 1). The volume of air sampled can be accurately measured using a flow meter. AAS is commonly employed for short-duration sampling, ranging from hours to days, making it suitable for monitoring daily or weekly variations [53]. However, AAS can also be utilized for long-term sampling, requiring hundreds or thousands of samples to obtain annual data. For instance, Hung et al. [54] utilized high-volume air samplers with a glass fiber filter and polyurethane foam (PUF) to monitor persistent organic pollutants (POPs) such as organochlorine pesticides and dichlorodiphenyltrichloroethanes in the Arctic region from 1993 to 2012. High-volume samplers effectively capture a higher amount of airborne pesticides compared to low-volume samplers [55]. However, the use of high-volume sampling techniques may introduce some minor errors, such as gaseous compounds being adsorbed on deposited particles or filters (blow-on), while volatile compounds may desorb from the filter (blow-off) [56]. To obtain a sufficient volume of pesticides in the air, Yusà et al. [55] suggested considering the sampling objective, sampler flow rate, and the analytical method’s limit of detection. In addition to high-volume samplers, diffusion denuder systems have been proposed for measuring semi-volatile organic compounds (SVOCs) such as polycyclic aromatic hydrocarbons (PAHs), polychlorinated biphenyls (PCBs), organochlorine pesticides (OCPs), or carbonyl compounds in the atmosphere [57,58]. Goriaux et al. [59] explained that high-volume samplers might not be suitable for determining SVOCs due to the degradation of particulate SVOCs on the filter, adsorption of gaseous SVOCs onto the filter, or reactions of collected SVOCs with atmospheric oxidizing species. Melymuk et al. [60] noted that the degradation of SVOCs is a natural process driven by interactions with atmospheric reactive species, including hydroxyl radicals, ozone, and NO_3_, as well as photolysis. It is essential to recognize that while a sampler should provide a snapshot of atmospheric SVOC concentrations, which may have already been affected by degradation, there is a possibility for degradation to persist within the sampler. This occurs as atmospheric reactive species are drawn into the sampling medium alongside the SVOCs. Within-sampler degradation represents an undesirable sampling artifact that can lead to the underestimation of ambient concentrations [61]. John et al. [62] also recommended using denuder systems as the preferred method for determining diffusion coefficients and monitoring the kinetic and dynamic behavior of flowing aerosols.

In terms of the filters employed in active air sampling, various materials have been used. Glass fiber filters [63] and quartz fiber filters [64] have been commonly utilized. According to Yusà et al. [55], the diameter of these filters typically ranges from 9 to 30 cm, depending on the specific sampling technique. 

When it comes to adsorbents for capturing gases, several materials have been employed. Polymeric phases such as polytetrafluoroethylene membranes (PTFE) [13], Tenax TA [65], XAD^®^-2 resins [66], XAD-4 [67], and PUF [67] have been used. Among these, XAD^®^-2 resins, which are hydrophobic copolymers of styrene-divinylbenzene resin, are widely preferred due to their high efficiency in trapping various types of herbicides, fungicides, and insecticides. PUF has also been extensively applied for monitoring OCPs and other pollutants such as PCBs, polybrominated diphenyl ethers (PBDEs), PAHs, and polychlorinated dibenzo-para-dioxins (PCDDs) [55,68,69,70]. Some studies have evaluated the trapping efficiency of mixed adsorbents. For instance, López et al. [71] used three different adsorbents (sandwich PUF-XAD2-PUF, XAD-2, and XAD-4) to capture 28 currently used airborne pesticides. However, Dobson et al. [72] compared the efficiency of XAD-2 with PUF, XAD-4 with PUF, XAD-2 with a PUF-XAD2-PUF sandwich, and PUF with a PUF-XAD4-PUF sandwich for trapping airborne pesticides. Their findings indicated that although the sandwiches exhibited higher efficiency compared to XAD-2 alone, they experienced a loss in pumping efficiency, possibly stemming from factors like elevated air resistance, clogging, or channeling within the adsorbent medium. 

### 3.2. Passive Sampling Applications and Limitations

**Figure 2:**
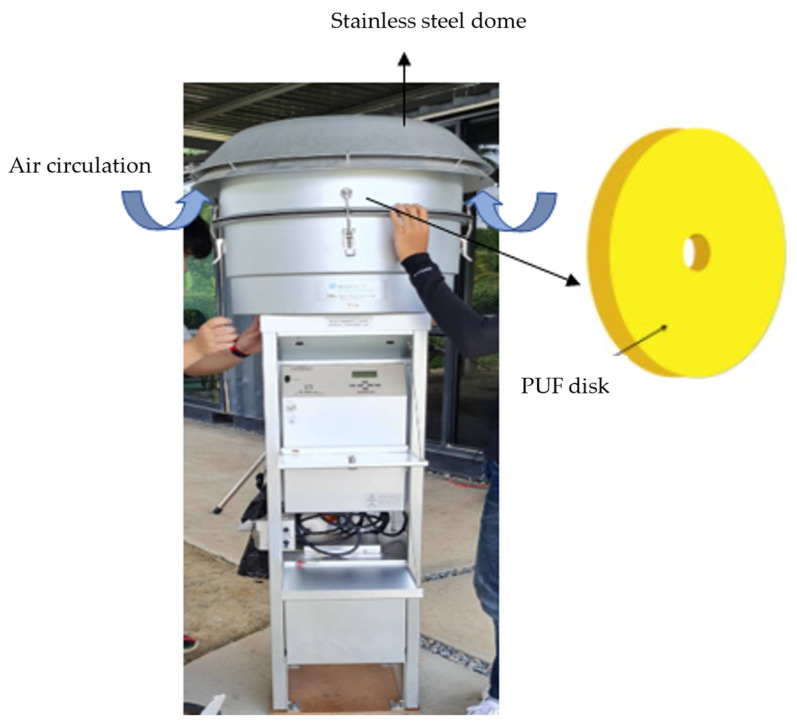
Diagram illustrating the passive air sampling technique.

Passive air sampling (PAS) is a technique that relies on the natural diffusion of gaseous pesticides through adsorbents, eliminating the need for a pump [73]. PAS typically operates at rates below 5 m^3^ day^−1^ [53]. A passive air sampler consists of a commercial accumulating medium with a high retention capacity for the target analytes (Figure 2). The adoption of PAS has gained global popularity over active air sampling (AAS) due to several reasons. Firstly, PAS offers convenience for integrative sampling in remote locations, as it does not require electricity [74,75]. Secondly, it allows for longer sampling durations, ranging from weeks to months, enabling the representation of monthly to yearly average exposure while minimizing spikes from episodic incidents [53,55,74]. Thirdly, PAS exhibits a broader range for sampling different types of pesticides, including both persistent and less persistent ones [32]. Furthermore, PAS is particularly suitable for monitoring indoor exposure to airborne POPs since it does not require the use of noisy pumps like AAS [76]. To obtain accurate results from PAS, it is essential to measure the correct air volume that passes through the sorbent during exposure [74]. Finally, PAS offers cost-effectiveness as fewer samples can effectively represent pollution sources and be analyzed, resulting in reduced expenses [53]. 

PAS generally employs various sorbent polymeric phases to capture pesticides. These include Tenax TA^®^, PUF disks, sorbent-impregnated PUF (SIP), semi-permeable membrane devices, and carbon-based foams [32,52,77,78,79,80,81]. PUF has been widely used since 2002, particularly for monitoring persistent compounds, but it has also been effective for sampling less persistent pesticides [32]. In response to the limitation of PUF in detecting glyphosate, TIEM Integrated Environmental Engineering from Germany has developed a polyester filter that effectively captures both glyphosate and aminomethylphosphonic acid in ambient air [38,82].

The choice of sorbent and housing design significantly affects the mass and type of collected particles. PUF and SIP can collect a representative portion of the particle phase, while XAD resins are suited for the gas phase [83]. PUF-based PAS has been widely utilized due to its high capacity for detecting atmospheric pollutants, simplicity in structure, cost-effectiveness, and ease of operation [79,84]. Typically, PUF disks are enclosed within two stainless steel bowls to regulate airflow and protect against precipitation and light [85]. 

PUF disks have been employed to collect various airborne pesticides, including POPs and SVOCs such as OCPs and OPs [86,87,88,89,90,91]. Nonetheless, Hayward et al. [92] noted that while the PUF-PAS might have reached equilibrium with the atmosphere during deployment, the average air concentrations over extended periods did not significantly differ from those determined by AAS. They also suggested that for evaluating long-term air concentration trends in a cost-effective manner, utilizing fewer samples, the preferred approach would involve year-long XAD-PAS deployments. Moreover, PUF’s low effective surface area and limited reusability pose challenges, especially when using PLE at high temperatures [52]. Despite the advantages of PAS, accurately measuring the specific volume of air passing through the adsorbent during exposure remains a drawback. To address this issue, Lévy et al. [52] recommended calculating the sampling rate based on field data. However, the variability in sampling rates can be attributed to several factors, including gas/particle partitioning, fluctuations in ambient air concentration, variations in ambient temperature and wind speed, and variations in particle concentration and size distribution in the air [93,94,95]. Therefore, Melymuk et al. [96] suggested three key approaches: (1) deriving sampling rates based on bulk concentrations rather than gas-phase concentrations alone, (2) utilizing continuous active air sampling, typically involving the collection of low-volume air samples, to capture bulk air concentrations (unattainable with depuration compounds), and (3) adopting homolog/KOA-specific sampling rates based on compound groupings, instead of compound/congener-specific rates or a single universal rate.

In addition to the existing sorbent materials, researchers have explored alternative options for monitoring air pollutants. One study by Pellicer-Castell et al. [97] introduced a novel passive air sampler using a sorbent based on UVM-7 mesoporous silica doped with Ti for detecting OCPs in occupational air. The results demonstrated that the Ti25-UVM-7 samplers were statistically comparable to the commonly used XAD-2 sampler. This new sampler offered an efficient, cost-effective, sustainable, and less solvent-consuming alternative for assessing occupational risk and measuring OCPs. 

Al-Alam et al. [78] compared the effectiveness of three carbon-based foams, namely, silicon carbide (SiC) foams, carbon nanotubes (CNTs), and graphite felts (GFs), for monitoring air pollution. The results indicated that the CNT foam and GF were inefficient in extracting PAHs, whereas the SiC foam exhibited superior properties and efficiency, making it the most effective passive sampler. Compared to the commonly used XAD^®^-2 resins, the SiC foam proved to be a plausible and efficient sorbent for PAS applications. This finding was further supported by a subsequent field study conducted by Al-Alam et al. [66], which confirmed the comparable efficiency of SiC foams and XAD^®^-2 resins. Moreover, the researchers explored the enhancement of SiC foam performance by grafting nitrogen-doped carbon (N-C) and nitrogen-doped carbon nanotubes (N-CNT) at various temperatures. The results revealed that the grafted SiC foams outperformed the conventional SiC foams, and had the potential to compete with the well-known XAD^®^-2 resins. Specifically, both 450 °C N-C SiC and 900 °C N-CNT SiC exhibited high potential for monitoring pesticides in the air. 

In a study conducted by Lévy et al. [52], the effectiveness of two distinct sorbents, namely, XAD^®^-2 resin and a carbon-based foam, was compared. The findings revealed that the utilization of the carbon-based foam as a passive sampler enabled the detection of a greater number of molecules compared to those captured by the XAD^®^-2 resin, which still remains the most efficient sampler for adsorbing highly volatile pesticides. Particularly, the nitrogen-doped carbon-silicon carbide foam (N-C@SiC) exhibited the highest efficiency in capturing particles and polar pesticides. This material allowed for a wider range of compounds to be sampled, including 2,4-MCPA, mecoprop-p, carbendazim, fluroxypyr, isoproturon, and others. The N-C@SiC foam offers a combination of advantages found in both PUF and XAD^®^-2 resin, including a high effective surface area, low pressure drops, and customizable surface chemical properties, making it a versatile material. Additionally, unlike XAD^®^-2 resin, the N-C@SiC foam can be reused after extracting the adsorbed pollutants [98]. Although the calculated sampling rates are somewhat limited, passive sampling utilizing the N-C@SiC foam, which possesses wider pores and is less hydrophobic, can serve as a viable alternative to currently employed adsorbents and active sampling methods for monitoring pesticides in the atmosphere [52].

When selecting appropriate adsorbents for PAS, it is crucial to consider the retention capacity and breakthrough for the target pesticides. The retention capacity helps identify any loss of pesticides during trapping in air sampling, while the breakthrough represents the maximum amount of pesticide that can be retained on a specific mass of adsorbent under given sampling conditions [15]. These factors play a significant role in determining the suitability and effectiveness of sorbent materials for PAS applications.

### 3.3. Evaluation of Sampling Techniques for Measuring Airborne Pesticides

**Figure 3:**
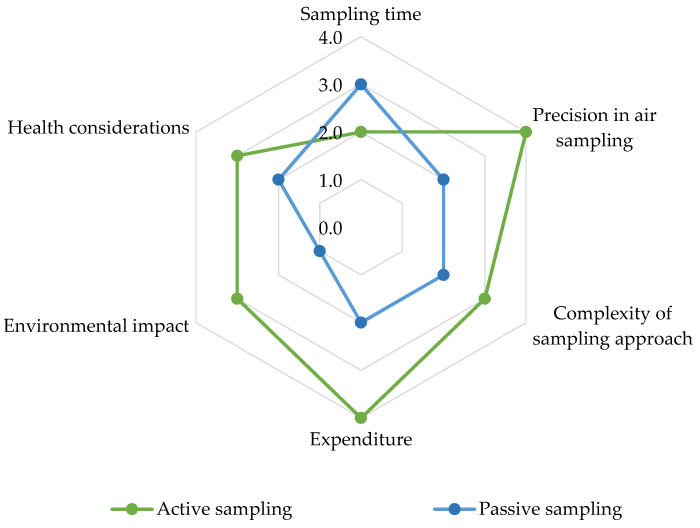
Comparative analysis of sampling techniques for airborne pesticide measurement. Level of impact: 1 = Low impact; 2 = Middle low impact; 3 = Middle high impact; 4 = High impact.

This review employs a spider chart to evaluate sampling techniques for measuring airborne pesticides, taking into consideration various factors (Figure 3). The assessment of each factor’s impact was conducted on a scale from 1 to 4, with 1 denoting a low impact, 2 indicating a moderate low impact, 3 suggesting a moderate high impact, and 4 representing a high impact. As known, active sampling methods involve deliberately directing airflow through a sampler, leading to swift sample collection. In contrast, passive sampling relies on natural diffusion, which extends the sampling duration. Nonetheless, it is important to consider that this extended exposure can cause degradation, affecting both the chemical compounds being measured and the sorbent material itself. As a result, compound-specific degradation may introduce variability in sampler calibrations [60]. Active sampling often achieves higher precision due to controlled airflow and consistent sample collection, while the precision of passive sampling is influenced by environmental factors like wind speed, temperature, and air concentrations. Various refinements in PAS have been introduced to address and control these factors, including the calibration of sampling rates, design modifications in sampler housing, and the use of performance reference compounds. However, despite these efforts, environmental variables can still introduce bias and errors in estimated air concentrations [60,99,100]. Moreover, active sampling methods can be more complex, involving components like pumps and power sources, whereas passive sampling techniques embrace simplicity with fewer components. In terms of costs, active sampling tends to incur greater expenses due to initial equipment investments, power requirements, and ongoing maintenance, including calibration. On the other hand, passive sampling is generally a cost-effective alternative, characterized by lower initial expenditures, simplified maintenance involving periodic sorbent material replacement, pre-calibrated options, and user-friendly operation. The environmental impact differs; active sampling may have a larger ecological footprint due to energy consumption and emissions, in contrast to the environmentally friendly nature of passive sampling. Lastly, it is worth noting that active air sampling methods, due to their electrical components, can present health risks if not adequately maintained and monitored for safety. In contrast, passive sampling, with minimal active components, tends to have fewer associated health concerns. These insights offer a comprehensive understanding of the strengths and limitations inherent in each sampling approach, facilitating well-informed decisions aligned with specific analysis requirements.

## 4. Emerging Trends in Pesticide Extraction Techniques: Enhancing Efficiency and Analytical Performance

### 4.1. Pesticide Extraction Techniques

The extraction step plays a crucial role in separating pesticide residues after sampling. The most commonly used technique for extracting target analytes from the samplers is liquid-solid extraction, with Soxhlet extraction (SE) being the prevailing method [101]. SE involves the use of a single solvent such as acetone or dichloromethane, or binary solvents like hexane-dichloromethane, dichloromethane-light petroleum, cyclohexane-acetone, or hexane-acetone [55]. However, traditional SE has certain drawbacks, including its time-consuming nature and the potential for environmental harm due to solvent release [102]. Moreover, this method requires a prolonged heating period, which can lead to thermal degradation of thermally labile compounds such as N-methylcarbamates, sulfonyl urea, and chlorophenoxy acid herbicides [55]. 

To address the concerns associated with SE and promote more environmentally friendly extraction techniques, alternative methods have been developed. These include liquid-solid extraction (LSE) [13], PLE [55,103], UAE [104], and MAE [105]. These techniques offer advantages over traditional SE, such as reduced extraction time, decreased solvent consumption, and enhanced extraction efficiency. LSE, PLE, UAE, and MAE have emerged as more environmentally friendly alternatives, contributing to the advancement of pesticide residue extraction methods. These approaches provide researchers with options that not only are efficient but also mitigate the potential negative impacts associated with traditional SE.

LSE is a commonly used technique for extracting pesticide residues but is often time-consuming, solvent-intensive, and laborious [106]. In contrast, alternative extraction methods offer advantages such as rapidity, automation, selectivity, and reduced solvent consumption, making them more environmentally friendly options [106]. Among these alternatives, PLE, also known as accelerated solvent extraction (ASE), has gained significant attention for the extraction of airborne pesticides due to its short extraction time, low solvent consumption, high contaminant yield, improved selectivity, and user-friendly system [107,108]. PLE involves using an extracting solvent to flush a solid or semi-solid sample under intense heat (50–200 °C) and high pressures (500–3000 psi) for a short duration (around 10 min) [55,109]. The efficiency of PLE depends on critical variables such as solvent selection, pressure, temperature, flush volume, extraction time, sorbent type, and the number of extraction cycles, necessitating optimization procedures [107]. 

Several studies have reported the successful application of PLE for the determination of airborne pesticides. For example, Coscollà et al. [110] employed PLE to extract various types of pesticides from fine airborne PM_2.5_ at trace levels. Mercier et al. [111] utilized PLE for the simultaneous analysis of SVOCs in indoor dust, encompassing musk fragrances, organochlorines (OCs), organophosphates (OPs), PAHs, PBDEs, PCBs, phthalates, and pyrethroids. Similarly, Kim et al. [108] developed analytical approaches for the examination of PAHs, OCPs, and PCBs in outdoor air using selective PLE. Rodrigues et al. [103] also employed PLE to quantify airborne pesticides in both active and passive samples as well as in dust. It is important to consider the potential degradation of chemical compounds during PLE due to the extreme conditions employed. A study by Lintelmann et al. [112] demonstrated the degradation of PAH quinones (OPAHs) during the PLE process, highlighting the need for careful evaluation and mitigation of compound degradation. Overall, PLE has emerged as a valuable technique for the extraction of airborne pesticides, offering efficient and environmentally friendly extraction with the potential for improved selectivity and reduced solvent consumption. The optimization of PLE parameters is crucial to ensure optimal extraction efficiency and minimize compound degradation.

UAE, also known as sonication, is an environmentally friendly technique used as an alternative method for extracting particle pollution. This method utilizes acoustic waves to generate cavitation bubbles, which enhance the solubility of analytes and the diffusion of solvents within the matrix [104]. UAE offers several advantages, including a significant reduction in extraction time, lower solvent usage, fewer opportunities for contamination and analyte losses, and the development of eco-friendly and cost-effective methods with increased productivity [109,113,114,115]. Aydin et al. [113] reported that using UAE for extracting PCBs, PAHs, and OCPs from ambient air could reduce solvent consumption by 80% and extraction time by 96% compared to traditional SE methods. 

One interesting aspect of UAE is that it only requires an ultrasound-producing device and adapted vessels to operate the system. In recent years, numerous studies have been conducted on the use of UAE for extracting airborne pesticides from environmental matrices. Dvoršćak et al. [116] extracted hexachlorobenzene, α-, β-, and γ-hexachlorocyclohexane, 4,4′-DDT, 4,4′-DDE, 4,4′-DDD, and 17 PCBs from PM_10_ and/or PM_2.5_ particle fractions in the northern part of Zagreb, Croatia, using a 1:1 acetone: n-hexane mixture in a UAE system. Beristain-Montiel et al. [104] utilized UAE micro-scale cells to extract organochlorine pesticides (OCls) and PBDEs from airborne PM. Additionally, Nascimento et al. [115] utilized UAE to determine the concentrations of OPs, pyrethroids, carbamates, and strobirulin in PM_2.5_ collected from a tropical coastal area in the Southern Hemisphere. 

It should be noted that UAE may be less precise than automated methods like PLE or MAE, especially when applied to matrices with strong interferences [117]. Furthermore, the sonication involved in UAE has the potential to damage sampling filters, which could result in the release of particles during the extraction process [109]. These factors should be taken into consideration when selecting the appropriate extraction method for airborne pesticide analysis. In summary, UAE offers a greener alternative for extracting pesticides from PM, providing advantages such as reduced extraction time, decreased solvent usage, and enhanced productivity. Despite its limitations in certain matrix types and the potential for filter damage, UAE has demonstrated successful applications in extracting airborne pesticides from solids and PM.

The extraction of pesticides and emerging pollutants using MAE or microwave-assisted solvent extraction has garnered significant interest in recent years. This approach utilizes microwave energy to facilitate the transfer of solutes from the matrix into the solvent. As a result, MAE offers comparable or higher extraction yields than other techniques, while requiring less time and solvent consumption [118]. Furthermore, MAE consumes less energy and generates fewer waste products, reducing environmental impact and human exposure [119,120]. There are two types of microwave systems commonly employed for extraction: “closed” extraction vessels for pressurized MAE, and “open” microwave ovens for focused MAE [118]. In pressurized MAE, the extraction process is conducted under both high pressure and high temperatures, while focused MAE is performed at atmospheric pressure to mitigate the risk of explosion. The maximum extraction temperature in MAE depends on the boiling point of the extraction solvents. However, Dean [121] highlighted certain limitations of focused MAE compared to pressurized MAE, such as the potential loss of volatile substances, risk of airborne transmission, inability to extract multiple samples simultaneously, and longer extraction times. 

Several studies have proposed the use of MAE for extracting pesticides from air samples. For instance, Coscollà et al. [48] employed MAE as a reliable and rapid procedure to determine pesticides in PM_2.5_ at trace levels. Naccarato et al. [122] utilized MAE to separate benzothiazoles (BTHs), benzotriazoles (BTRs), and benzenesulfonamides (BSAs) in airborne PM_10_ samples. Overall, MAE offers a promising extraction method for pesticides and emerging pollutants, providing advantages in terms of efficiency, reduced solvent consumption, and environmental impact. However, to choose between pressurized MAE and focused MAE, one should consider the specific requirements of the analysis, including the target compounds and sample characteristics, to ensure optimal extraction performance.

### 4.2. Analytical Performance of Pesticide Extraction Methods

**Figure 4:**
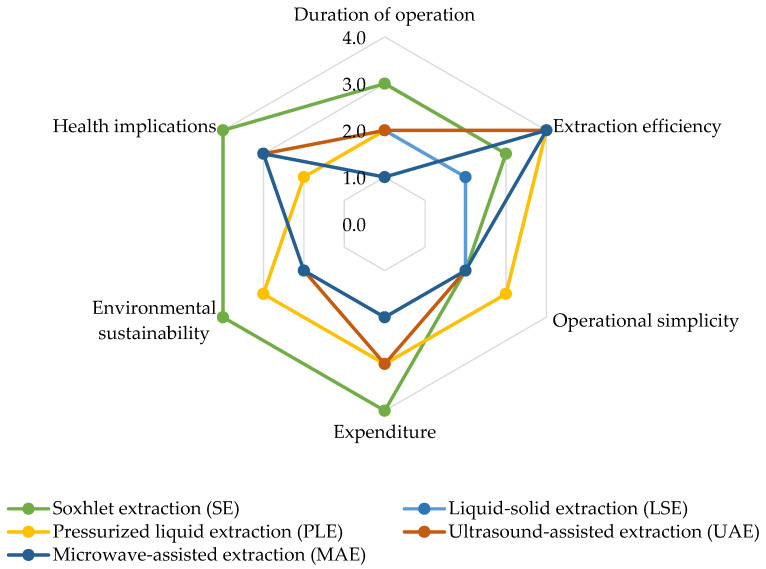
Comparative evaluation of extraction methods for airborne pesticide analysis. Level of impact: 1 = Low impact; 2 = Middle low impact; 3 = Middle high impact; 4 = High impact.

When conducting a comparative assessment of various extraction methods for analyzing airborne pesticides through the utilization of a spider chart (Figure 4), several crucial factors emerge. For instance, the operational time varies, with SE entailing time-consuming processes due to prolonged extraction cycles. In contrast, methods like PLE and MAE expedite procedures by capitalizing on elevated temperature and pressure or microwave heating. Extraction efficiency also displays variability: SE and PLE excel in efficiency, particularly for heat-sensitive compounds in the case of PLE, while UAE and MAE ensure effectiveness through improved mass transfer or swift and uniform heating. The spectrum of operational simplicity ranges from complexity with SE to relative simplicity with UAE and MAE. 

In terms of expenditure, SE and LSE may be cost-intensive due to equipment and solvent consumption, while PLE necessitates initial investment in pressurized equipment, and UAE and MAE involve moderate costs with specialized equipment. Environmental sustainability tips in favor of PLE, benefiting from reduced solvent usage, while health implications underscore that PLE and UAE offer comparatively safer alternatives due to minimized solvent exposure through enclosed systems or optimized conditions. It is essential to recognize that the method’s efficacy can be shaped by compound characteristics, sample matrix, equipment quality, and parameter optimization, underscoring the need to select the most fitting method in line with distinct study requisites and limitations.

## 5. Advancements in Pesticide Analytical Methods: Exploring Classical and Recent Technologies

Airborne pesticides can be identified and quantified using two primary chromatographic techniques: high-performance liquid chromatography (HPLC) and GC. The selection between these methods depends on factors such as the volume of extracted samples and the specific clean-up procedure required.

### 5.1. Advances in GC for Pesticide Detection in Air

The measurement of atmospheric pesticides poses a significant challenge, particularly when dealing with concentrations below 2 ng/m^3^ [74]. In both AAS and PAS, an extraction process is necessary to release the trapped pesticides from the media. Two common techniques for extraction are organic solvent desorption and thermal desorption (TD). The organic solvent method involves multiple extraction and concentration steps, resulting in higher uncertainty and a more time-consuming process [74]. In contrast, TD eliminates the need for a concentration step, and it can be coupled with GC-MS to achieve lower quantification limits compared to organic solvent extraction. GC is widely utilized for pesticide measurements in combination with various element-specific detectors, such as atomic emission (AED), electron capture (ECD), sulfur chemiluminescence (SCD), nitrogen-phosphorus detection (NPD), and MS. 

GC-ECD is commonly used for the analysis of OCPs in food and environmental samples due to its high separation efficiency, sensitivity, and cost-effectiveness [123,124,125]. Non-polar or semi-polar columns with dimensions of 30 m × 0.32 mm ID × 0.25 mm film thickness are typically employed, and helium is commonly used as the carrier gas [55]. However, it is important to note that GC-ECD is particularly well suited for the analysis of thermally stable compounds due to its thermal desorption capabilities. Given this consideration, Liu et al. [125] recommended using GC-negative chemical ionization-mass spectrometry (GC-NCI-MS) instead of GC-ECD for analyzing OCPs and hexabromobiphenyls (HBBs) in atmospheric PM and soil samples. They found that GC-NCI-MS provided better sensitivity and robustness compared to GC-ECD. This recommendation aligns with Garrido Frenich et al. [123], who highlighted the interferences that can occur in GC-ECD analysis, suggesting that they can be minimized or avoided by using the GC-MS/MS method. Overall, due to the complex nature of environmental matrices and the need to detect compounds at extremely low levels, MS has emerged as the preferred option for element-selective detection following GC separation [126]. MS offers enhanced sensitivity and robustness, making it a valuable tool for the analysis of complex environmental samples containing organometallic compounds at increasingly low concentrations.

Nowadays, GC-MS is the predominant technique used for determining pesticides in the air, primarily due to the volatility of most compounds [127]. Capillary columns with various trade names, such as MDN-5, DB-5, TR-5MS, SGE-BPX5, or V5-MS, are commonly employed [9,15,128,129,130]. Helium gas with a purity of 99.99% or argon C50 gas with a purity of 99.99% is typically used as the carrier gas [128]. In GC-MS analysis, the major application for the MS analyzer is the use of quadrupole in selected ion monitoring (SIM) mode, which offers higher sensitivity compared to the full scan mode [55]. The MS analyzer is primarily operated in the electron ionization (EI) mode, especially in multi-residue analysis. 

Pesticide quantification is based on the GC-MS peak area, and external calibration curves are generated by directly injecting analytical standards of the target pesticides. However, the use of tandem mass spectrometry (MS/MS) has recently been proposed as a valuable tool due to its increased selectivity and reduced mass spectral noise [131]. Lee and Jo [132] and Wu [133] emphasized the excellent selectivity and sensitivity provided by GC-MS/MS with a triple quadrupole (QqQ) analyzer. It can be operated in multiple reaction monitoring (SRM) mode, enabling more reliable identification and quantification of target analytes. Coscollà et al. [128] found that although GC-MS/MS-QqQ was a highly effective tool for multi-residue analysis of airborne pesticides in PM_10_, the use of matrix-matched standard calibration methodology was necessary for more accurate quantitative analysis. GC-MS is also coupled with a thermal desorption (TD) unit for simultaneous extraction and concentration of trapped pesticides. Decuq et al. [74] recently introduced a multi-residue analysis technique using TD-GC-MS to quantify pesticides in both the atmosphere and rainwater within a single process. This approach has shown promising results in the field of pesticide analysis.

To ensure satisfactory sensitivity for each pesticide, the precise optimization of MS/MS variables is required. The initial step involves selecting the parent ions from each pesticide using a full scanning spectra mode. Subsequently, the precursor ions are accumulated and isolated in the ion trap, followed by fragmentation through collision-induced dissociation [55]. Among the resulting product ions for each congener, the two most prominent ones are selected. The optimization process can be carried out using either the approach of changing one factor at a time or the design of experiment procedure [120]. This optimization ensures that the MS/MS settings are fine-tuned for optimal sensitivity and the accurate identification of target pesticides.

Some researchers have highlighted the use of GC-MS/MS employing ion trap (IT) instruments, known as GC–IT–MS/MS, for the analysis of various pesticides due to its superior sensitivity and specificity. Scheyer et al. [134] developed a multi-residue method using GC–IT-MS/MS to analyze 27 pesticides in atmospheric samples, including both particle and gas phases. However, certain pesticides such as phenoxy acids and bromoxynil required a derivatization step using pentafluorobenzylbromide. López et al. [15] also utilized GC–IT–MS/MS to detect specific pesticides like diphenylamine, pyrimethanil, bifenthrin, lambda-cyhalothrin, permethrin, and cypermethrin in indoor air samples. Another powerful detector, ICP-MS/MS (inductively coupled plasma-tandem mass spectrometer), offers robust and sensitive analysis for heavily interfered elements, particularly for volatile organometallic contaminants. However, the application of GC-ICP-MS for the determination of relevant pesticides is currently limited. Somoano-Blanco et al. [135] measured PCBs using GC-ICP-MS and found the sensitivity obtained to be insufficient for the reliable quantification of the target PCBs in samples. So far, this detector has not been extensively employed for pesticide analysis in air samples. Recently, Décuq et al. [74] demonstrated that TD-GC-MS is an excellent technique for detecting and quantifying pesticide samples in both atmospheric and rainwater samples, providing accurate results with minimal analyte amounts. This method offers a promising approach for the efficient analysis of pesticides in environmental matrices.

### 5.2. Advances in LC for Pesticide Detection in Air

GC-MS in SIM mode and GC-MS/MS-QqQ are commonly used for GC-amenable pesticides. However, for non-GC amenable pesticides, such as polar, non-volatile, and thermolabile compounds like herbicides, carbamates, triazines, phenoxy acids, or neonicotinoids, HPLC has been proposed as an alternative method. HPLC is suitable for the separation and quantification of these pesticides, especially when they require derivatization to enhance volatility, thermal stability, and sensitivity, or to address the limitations of GC-MS methods. Despite the prevalence of GC-MS techniques, there are limited studies that have applied HPLC methods for pesticide analysis in ambient air. Examples include Li et al. [136] employing HPLC with fluorescence detection after online derivatization to analyze nine carbamates collected from air samples using OSHA versatile sampler (OVS) tubes. Coscollà et al. [108] developed an approach involving MAE followed by direct injection into LC-MS/MS for the extraction and analysis of currently used pesticides in fine airborne PM_2.5_.

Raina and Hall [137] utilized LC-MS/MS with electrospray ionization in the positive mode (ESI+) to determine OPs and their degradation products in air samples, finding better sensitivity in ESI+ compared to the atmospheric pressure chemical ionization (APCI) mode. This could imply that while GC-MS methods dominate the analysis of GC-amenable pesticides in air, HPLC techniques have been proposed for the quantification of non-GC amenable pesticides, particularly those with polar or thermolabile characteristics. Although fewer studies have employed HPLC for pesticide analysis in ambient air, examples demonstrate its applicability for specific pesticide classes and trace-level measurements [55,138].

MS/MS techniques have certain limitations highlighted by Rajski et al. [139], including the need for the optimization of acquisition parameters for each targeted compound, the limited number of compounds that can be identified, and the inability to detect compounds not included in the monitored analyte list. This implies that MS/MS techniques may overlook compounds present in the sample but not specifically targeted. To overcome these limitations, LC-HRMS has emerged as an alternative approach. The key advantage of LC-HRMS, particularly using instruments such as time-of-flight (TOF) and Orbitrap, is the ability to acquire data for an unlimited number of compounds through accurate mass measurements (1–5 ppm) and high resolving power (25,000–50,000 FWHM). This allows for comprehensive screening and analysis of various species [140,141]. Additionally, LC-HRMS exhibits improved sensitivity compared to LC-MS/MS [142]. López et al. [71,141] proposed a comprehensive strategy for the retrospective analysis of pesticide metabolites in ambient air using LC-HRMS. Their findings demonstrated the potential of this strategy in providing valuable insights and clarifying unknown metabolites. However, the identification of unknown metabolite compounds requires significant computational efforts.

### 5.3. Other Advances in Pesticide Detection Methods

IMS has recently gained significant prominence as a robust separation technique owing to its unique design, high sensitivity, rapid response time, operation at ambient pressure, and capability to effectively separate isomeric compounds. This versatile technique finds applications in diverse fields such as chemical weapons detection, explosives analysis, pharmaceutical screening, and environmental monitoring [143]. In IMS, gas-phase ions are generated by ionizing neutral molecules, and subsequently, they are separated based on their distinct velocities within an electric field before quantification. The separation efficiency is influenced by factors such as temperature, pressure, and the molecular properties of the drift gas. Building upon these principles, Gallart-Mateu et al. [13] developed an innovative IMS method for the detection of airborne pesticides. Both negative and positive IMS modes were employed to analyze different types of pesticides. The researchers suggested that this proposed method holds immense potential as a rapid, efficient, sensitive, and environmentally friendly analytical tool for the identification and quantification of pesticides in both indoor and outdoor air samples.

Attenuated total reflectance–Fourier-transform infrared spectroscopy (ATR–FTIR) spectroscopy is a widely utilized technique for the chemical characterization of environmental samples [144,145]. ATR, as a sampling mode, enhances the FTIR signal obtained from the surfaces of samples, making it a promising tool for detecting organic pesticides and hazardous mineral compounds such as asbestos, which is a re-emerging contaminant in environmental matrices [14]. While ATR-FTIR offers rapid and non-invasive analysis, with improved reproducibility compared to traditional analytical methods, it has limitations in the molecular-level identification of contaminants of emerging concern. To address this challenge, a complementary spectroscopic analysis data of non-target analytes is recommended. Sreejith et al. [146] employed ATR–FTIR spectroscopy in combination with liquid chromatography coupled with quadrupole time-of-flight mass spectrometry (LC-Q-ToF-MS) to screen indoor dust collected from a tropical coastal metropolitan area. The ATR–FTIR results revealed the presence of re-emerging contaminants such as aldehydes, anhydrides, carboxylic acids, esters, sulphonic acids, and asbestos. In contrast, LC-Q-ToF-MS analysis allowed for the identification of plasticizers, plasticizer metabolites, photoinitiators, personal care products, pharmaceutical intermediates, surfactants, and pesticides. It is important to highlight that ATR-FTIR can serve as a complementary technique to LC-Q-ToF-MS for the determination and characterization of unknown indoor pollutants [146].

Synchrotron radiation–attenuated total reflectance–Fourier-transform infrared spectroscopy (SR-ATR-FTIR) represents a sophisticated analytical approach leveraging synchrotron light’s unique qualities [147,148]. Unlike traditional FTIR, it offers brightness, spatial precision, and energy versatility, with distinct advantages for airborne pesticide analysis in environmental samples. This technique requires the preliminary processing or extraction of environmental components like soil, water, or plant tissue. With SR-FTIR, the interaction between emitted infrared radiation and the sample generates molecular fingerprints. Exposure to potent synchrotron radiation enables the selective absorption of IR light by pesticide functional groups, yielding specific spectral peaks. The exceptional resolution capability of SR-ATR-FTIR facilitates microscale exploration, uncovering pesticide traces undetected by conventional methods like GC-MS and LC-MS. Spectral examination aids in identifying pesticide-specific absorption peaks, enabling the qualitative and quantitative assessment of residues using reference spectra. Overall, SR-ATR-FTIR stands as a powerful tool for intricate airborne pesticide analysis within complex environmental settings.

High-pressure anion exchange chromatography (HPAEC) is another chromatographic technique that utilizes an anion exchange column to separate analytes based on their ionic properties. Pesticides with anionic moieties, such as carboxylates or sulfonates, can be separated and retained on the column while other interfering compounds are removed. Recently, Feltracco et al. [149] used the HPAEC-MS/MS method for investigating the presence of airborne polar pesticides at two sites in north-eastern Italy. Fourteen polar pesticides (glyphosate, AMPA, N-acetyl AMPA, glufosinate, N-acetyl glufosinate, MPPA, FOS-Al, phosphonic acid, ethephon, HEPA, maleic hydrazide, cyanuric acid, chlorate, and perchlorate) in PM_10_ aerosol samples were found. However, HPAEC-MS/MS exhibits exceptional selectivity specifically for the analysis of pesticides containing anionic moieties. Developing an HPAEC-MS/MS method can be complex and time-consuming as it requires the optimization of chromatographic conditions, mobile phases, and MS/MS parameters to achieve the optimal separation and detection of the target analytes. Moreover, air samples can contain complex matrices with potential interferences that may affect the chromatographic separation or ionization efficiency. Matrix effects can impact the accuracy and reliability of quantification results, requiring careful sample preparation and matrix-matched calibration.

### 5.4. Analytical Performance of Pesticide Detection Techniques

**Figure 5:**
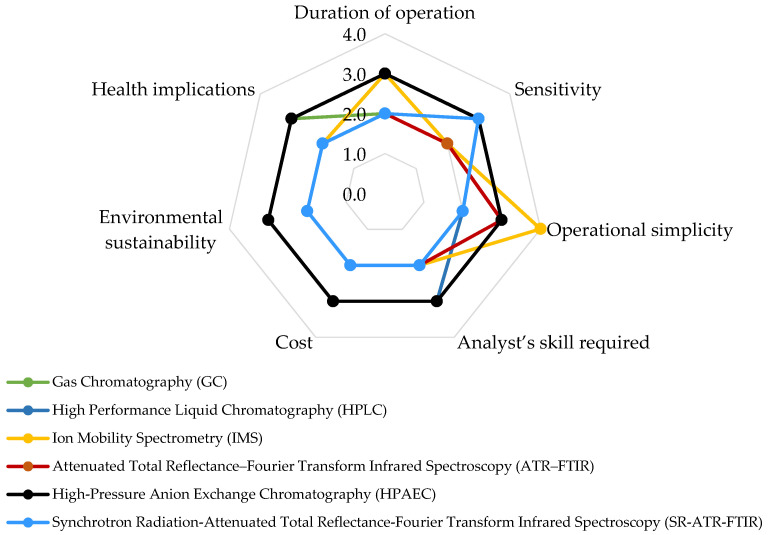
Comparative assessment of analytical techniques for airborne pesticide analysis. Level of impact: 1 = Low impact; 2 = Middle low impact; 3 = Middle high impact; 4 = High impact.

When employing a spider chart to assess various techniques for measuring airborne pesticides across different factors (Figure 5), several pivotal considerations become apparent. In terms of operational time, GC techniques exhibit variations depending on the specific method utilized (e.g., GC-MS, GC-FID). This encompasses stages such as sample preparation, injection, column separation, and detection, collectively contributing to a distinct timeframe. Similarly, the HPLC method requires several sequential processes, making it relatively time-consuming. In contrast, IMS expedites analyses due to its rapid ion mobility separation process. ATR–FTIR and SR-ATR-FTIR offer quicker analysis due to minimal sample preparation and the efficiency of FTIR techniques. Likewise, leveraging synchrotron sources for rapid scanning enables time-resolved studies, facilitating insights into pesticide degradation and transformation in the environment. HPAEC involves multiple steps similar to GC and HPLC, contributing to a moderately paced operational timeframe.

Regarding sensitivity, both GC and HPLC methodologies are renowned for their exceptional sensitivity, enabling the detection of trace compounds, including airborne pesticides, within complex matrices. Similarly, IMS also demonstrates sensitivity to specific compound classes, although it might not reach the same level as chromatography-based methods. ATR–FTIR recognizes functional groups and chemical bonds, albeit sensitivity toward specific pesticides may exhibit variance. HPAEC’s sensitivity extends to anions, encompassing selective pesticides and aligning with specific applications. SR-ATR-FTIR offers high sensitivity, making it suitable for detecting trace amounts of pesticide residues.

In assessing operational simplicity, GC and HPLC demand adept analysts and intricate hardware, diminishing their operational ease. IMS stands out as comparatively user-friendly, necessitating minimal analyst expertise when compared to chromatographic methods. ATR–FTIR demonstrates relative straightforwardness with minimal sample preparation and swift analysis. HPAEC introduces a degree of complexity, manageable with proper training. Meanwhile, SR-ATR-FTIR demands a certain level of skill but offers a harmonious blend of advanced capabilities and user-friendliness.

When evaluating the requisite analyst skill, GC and HPLC demand proficient analysts for meticulous method development, sample preparation, and data interpretation. IMS calls for less analyst expertise in comparison to intricate chromatographic approaches. ATR–FTIR and SR-ATR-FTIR mandate basic training for operational competence and spectral interpretation. HPAEC, on the other hand, necessitates adept analysts, proficient in method development, sample preparation, and data interpretation.

In terms of cost analysis, GC and HPLC instruments, along with their consumables and maintenance, incur moderate expenses. IMS instruments are often affordable, with generally lower operational costs in comparison to chromatography. ATR–FTIR instruments fall within a moderate cost range, with routine analyses proving cost-effective. For HPAEC, instruments and consumables hold moderate costs, justified by the technique’s specificity. On the other hand, SR-ATR-FTIR entails higher costs due to synchrotron access and equipment requirements, but its capabilities substantiate the investment. 

From an environmental sustainability standpoint, GC and HPLC utilize solvents that can potentially impact the environment. However, ongoing efforts are directed towards adopting greener practices to mitigate these effects. In contrast, IMS stands out for its reduced solvent usage and minimized waste generation, which contribute to a more environmentally sustainable approach. Both ATR–FTIR and SR-ATR-FTIR require minimal reagents and generate limited waste, further reinforcing their positive environmental profile. 

Turning to health implications, GC and HPLC demand the vigilant handling of solvents and analytes to mitigate potential health risks associated with hazardous substances. On the other hand, IMS involves fewer harmful chemicals, alleviating health concerns for analysts. The ATR–FTIR and SR-ATR-FTIR methods entail minimal exposure to hazardous chemicals, which significantly reduces health risks for practitioners. HPAEC necessitates the proper management of solvents, reagents, and analytes to effectively minimize potential health hazards. This comprehensive evaluation highlights the significance of considering environmental sustainability and health aspects when choosing among these analytical techniques.

## 6. Future Directions and Opportunities in Pesticide Detection and Monitoring

The emission of pesticides during and after their application has a significant impact on both indoor and outdoor air quality, as well as other environmental compartments. Given the potential toxicities and the prolonged exposure of humans to pesticides, it is crucial to assess the risk associated with airborne pesticides by measuring their concentrations. This assessment can be accomplished through either active or passive sampling methods, depending on the approach used for sample collection. Active samplers find widespread use across different methodologies; nonetheless, they are characterized by higher complexity and costs in comparison to passive samplers. In contrast, passive air sampling stands out for its inherent accuracy, capitalizing on the natural phenomenon of diffusion. This approach is especially well suited for extended, long-term assessments. However, it is essential to acknowledge that diffusive air sampling techniques are inherently restricted to gases and vapors, with their uptake rate being fixed by design. Looking toward future directions, there is an evident opportunity to further enhance the usability and cost-effectiveness of active sampling methods for measuring airborne pesticides. Innovations in miniaturization and automation technologies could lead to more user-friendly and compact active sampling devices, lowering barriers to deployment and reducing overall costs. 

Research efforts could be directed towards improving the sorbent materials used in passive sampling, increasing their selectivity and capacity to capture an even wider range of pesticide compounds. For instance, the development of novel passive sampling devices utilizing advanced sorbent materials with high affinity for diverse pesticides could lead to improved accuracy in capturing a wider array of compounds present in the air. Furthermore, the development of hybrid sampling approaches that integrate both active and passive techniques could provide a more comprehensive view of airborne pesticide concentrations, combining the benefits of precision and extended deployment. These advancements will be crucial in expanding capabilities to monitor and assess airborne pesticide contamination, contributing to more informed environmental management strategies and policies.

In the realm of isolating specific compounds from airborne samples, the prevailing approach is SPE, which utilizes various solvents. However, innovative multi-residue methods like UAE or MAE have emerged as alternatives. These methods aim to curtail the use of harmful solvents, reduce extraction time, and lower overall costs. Nonetheless, they can pose challenges due to the need for specialized and expensive equipment. Looking ahead, a pressing requirement exists for the ongoing development of analytical instruments that are both cost-effective and high-performing in the realm of environmental analysis. This advancement will effectively tackle the issue of detecting and quantifying pesticides in air samples, paying heed to matrix characteristics and the desired concentration levels of the target compounds.

Presently, GC and LC coupled with MS, such as LC-MS, LC-MS-MS, and GC-MS, are widely employed for the quantitative analysis of pesticides. Nevertheless, these methods can be intricate, time-intensive, and financially demanding. Their complex operational nature renders them more suitable for on-site applications where skilled personnel can navigate their intricacies. However, there remains ample opportunity for transformative advancements. Emerging detection approaches, such as IMS, ATR-FTIR, and SR-ATR-FTIR, offer accelerated, functional, sensitive, and environmentally conscious analytical tools for pesticide detection in air samples. These innovative methods show potential for expedited research and real-world application in pesticide analysis, hinting at a promising trajectory for the field. In forging this path forward, the development of user-friendly, cost-effective, and rapid techniques will play a pivotal role in enhancing our ability to monitor and understand airborne pesticide contamination, thereby strengthening environmental protection strategies and fostering a healthier ecosystem for the future.

## 7. Conclusions

This review delivers several pivotal insights into the realm of airborne pesticide sampling and analysis. Firstly, it underscores the critical importance of effectively examining and quantifying airborne pesticides, offering a robust foundation for researchers and environmental practitioners alike. Secondly, it serves as a practical and comprehensive guide for those navigating the selection of sampling methods and analytical techniques. By carefully considering key factors such as operational time, sensitivity, cost, environmental impact, and health concerns, this review aids in the informed decision-making process. Lastly, this review empowers researchers, professionals, and policy makers with the knowledge and tools needed to advance an understanding of airborne pesticide behavior, leading to more effective management and regulatory strategies for pesticide usage and bolstering environmental protection endeavors.

## Figures and Tables

**Table 1:** Types and concentrations of pesticides in indoor and outdoor air.

Pesticide	Name	Indoor Air Concentration Range (ng m^−3^)	Outdoor Air Concentration Range (ng m^−3^)	Investigated Country	References
Organochlorine	Chlordane	3.50–4.80	0.01–0.02	USA, South Africa	[19,20]
	4,4′-DDE	1.20–190	0.002–25.60	Nepal, USA, China, Czech Republic	[19,21,22,23]
	4,4′-DDD	ND ^1^–5.00	0.011–154	China, Czech Republic, USA, South Africa	[20,22,23,24]
	HCB	0.16–1.80	ND–0.43	Czech Republic, Canada, China	[23,24,25,26]
	HCH	0.05–7.82	0.00012–0.0184	Czech Republic, Canada, China, South Africa	[20,24,25,26]
	Heptachlor	5.00	0.00065–0.0015	USA, South Africa	[19,20]
	Endosulfan	0.3–0.8	0.00009–81.31	France, Nepal, Pakistan, China	[21,22,26,27,28,29]
	Mirex	ND–0.00477	ND–9.94	USA, South Africa	[20,24]
	Lindane	1.4–11.8	NI ^2^	France	[27]
	Aldrin	NI	0.36–1.18	China	[22]
	4,4′-methoxychlor	NI	0.00040–0.00883	China	[26]
	Oxychlordane	NI	0.00048	South Africa	[20]
Organophosphate	Chlorpyriphos	0.4–83.4	0.1–36.1	USA, Spain, Costa Rica, Czech Republic, South Africa	[20,30,31,32,33,34]
	Diazinon	1.0–427.5	0.28–1.49	USA, France, South Africa	[19,20,28,30]
	Malathion	0.2–16.1	0.010–1.0	France, USA, Spain, Czech Republic, South Africa	[20,27,30,31,33]
	Parathion-methyl	14.3	NI	France	[27]
	Dichlorvos	22.9–53,000	NI	France, South Korea	[27,35]
	Dimethoate	NI	0.1–1.0	South Africa	[20,34]
	Ethoprophos	NI	0.21–0.48	France	[28]
Carbamates	Carbaryl	NI	1.30	South Africa	[20]
Acetamides	Metazachlor	NI	0.0092–3.13	25 member states of the European Union (EU-25) ^3^, South Africa	[20,36]
Triazinones/ Triazines/ Triazoles	Metribuzin	NI	0.03	South Africa	[20]
	Atrazine	NI	0.04	South Africa	[20]
	Simazine	NI	0.88	South Africa	[20]
	Terbuthylazine	NI	0.79	South Africa	[20]
	Propiconazole	NI	0.08	South Africa	[20]
	Tebuconazole	NI	0.43–22.2	South Africa	[20,34]
Herbicide	Alachlor	NI	0.12–6.03	France	[28]
	Alconifen	NI	0.23–4.15	25 member states of the European Union (EU-25)	[36]
	Diuron	NI	0.12	South Africa	[20]
	Glyphosate	NI	0.18–510.0	USA, Malaysia, France	[37,38,39]
	Trifluralin	NI	0.12–40.74	25 member states of the European Union (EU-25)	[36]
Fungicide	Captan	NI	1.19–67.62	25 member states of the European Union (EU-25)	[28,36]
	Chlorothalonil	NI	0.11–107.93	France	[28]
	Carbendazim	NI	0.028	Spain	[31]
	Epoxiconazole	NI	0.12–3.99	France	[28]
	Folpet	NI	7.91–82.22	25 member states of the European Union (EU-25)	[36]
	Tebuconazole	NI	22.2	Czech Republic	[33]

^1^ ND = Not detected. ^2^ NI = No information. ^3^ EU-25 [36]: Austria, Belgium, Bulgaria, Czech Republic, Denmark, Estonia, Finland, France, Germany, Greece, Hungary, Ireland, Italy, Latvia, Lithuania, Luxembourg, Netherlands, Poland, Portugal, Romania, Slovakia, Slovenia, Spain, Sweden, United Kingdom.

## Data Availability

Data will be made available on request.
